# Projecting the Burden of Chronic Kidney Disease in a Developed Country and Its Implications on Public Health

**DOI:** 10.1155/2018/5196285

**Published:** 2018-07-04

**Authors:** L. Y. Wong, A. S. T. Liew, W. T. Weng, C. K. Lim, A. Vathsala, M. P. H. S. Toh

**Affiliations:** ^1^Chronic Disease Epidemiology, Population Health, National Healthcare Group, Singapore; ^2^Renal Medicine, Tan Tock Seng Hospital, Singapore; ^3^Clinical Services, National Healthcare Group Polyclinics, Singapore; ^4^Nephrology, National University Hospital, Singapore; ^5^Population Health, National Healthcare Group, Singapore

## Abstract

**Background:**

Chronic Kidney Disease (CKD) is a major public health problem worldwide. There is limited literature on a model to project the number of people with CKD. This study projects the number of residents with CKD in Singapore by 2035 using a Markov model.

**Methods:**

A Markov model with nine mutually exclusive health states was developed according to the clinical course of CKD, based on a discrete time interval of 1 year. The model simulated the transition of cohorts across different health states from 2007 to 2035 using prevalence, incidence, mortality, disease transition, and disease detection rates.

**Results:**

From 2007 to 2035, the number of residents with CKD is projected to increase from 316,521 to 887,870 and the prevalence from 12.2% to 24.3%. Patients with CKD stages 1-2 constituted the largest proportion. The proportion of undiagnosed cases will decline from 72.1% to 56.4%, resulting from faster progression to higher CKD stages and its eventual detection.

**Conclusion:**

By 2035, about one-quarter of the Singapore residents are expected to have CKD. National policies need to focus on primary disease prevention and early disease detection to avoid delayed treatment of CKD which eventually leads to end-stage renal disease.

## 1. Introduction

Chronic Kidney Disease (CKD) is a major public health problem worldwide. The prevalence of CKD in Singapore was reported to be 15.6% [1] in 2007. This was considered excessive compared to countries with higher incidence of end-stage renal disease (ESRD) such as USA [2], Korea [3], Japan [4], and Taiwan [5]. Although the definition of CKD used in the estimation of prevalence for these countries was inconsistent and made cross-country comparison challenging, the prevalence of CKD in Singapore remained relatively high even after adjustments were made to allow for comparison across these countries [2–5].

Previous literature [6] reported high prevalence of cardiovascular diseases in individuals with CKD, and likewise, CKD had been recognized as an independent risk factor of cardiovascular disease outcomes. CKD is associated with elevated risks of all-cause mortality and increased healthcare utilization [7–9]. The hazard ratios of death among individuals with CKD stages 3-5 vis-à-vis those without CKD ranged between 1.2 and 5.9 [7, 10]. In addition, individuals with CKD were 1.6 - 2.2 times more likely to be hospitalized [9]. Consequently, the unadjusted annual incremental direct all-cause healthcare costs associated with CKD among cohorts with (a) diabetes only, (b) hypertension only, and (c) both diabetes and hypertension were USD11,814, USD8,412, and USD10,625, respectively [11]. Patients with CKD were also reported to have compromised quality of life (QoL), with progressively lower QoL scores as CKD advances [12]. Thus, the burden of CKD to both the healthcare providers and patients is heavy and overwhelming.

Notwithstanding that earlier CKD stages, if treated early, may prevent the progression to ESRD [13, 14], the rate of diagnosis and public awareness of CKD remain low [2, 5, 15, 16]. Many people with CKD present in late stages with some presenting only during ESRD requiring dialysis or renal transplantation. From 2011 to 2014, Singapore had consistently ranked as one of the top five countries with the highest ESRD incidence rates in the world [17]. Consequently, as the prevalence of CKD increases, the impact on the ESRD burden will become increasingly significant. By 2030, 20.5% of Singapore's population is estimated to be elderly (age more than 65 years) [18]. With an ageing population, the prevalence of CKD is expected to increase considerably as kidney function declines even with normal ageing [19]. Therefore, projecting the future prevalence of CKD, especially for the population in whom the condition is undiagnosed, would quantify the magnitude of its burden with insights on disease progression. This can influence policy-making to address public health concerns and develop preventive interventions to retard CKD progression.

Currently, there is limited literature on a model to project the number of people with CKD and undiagnosed cases. The aim of this study is to project the number of Singapore residents who are aged 21+ years and have CKD in 2035 using a Markov model.

## 2. Methods

### 2.1. Markov Model and Health States

A Markov model, which assumes that an individual is always in one of a finite number of discrete health states, is commonly used to project the number of persons with mutually exclusive health states [20–22]. It offers a dynamic forecasting approach by simulating the progression of individuals from one health state to another at discrete time intervals (i.e., cycles) based on time-dependent transition probabilities,* Pij (0, t)*, which is the probability of an individual who is in state i at time 0, who will transit to be in state j at time t.

**Figure 1:**
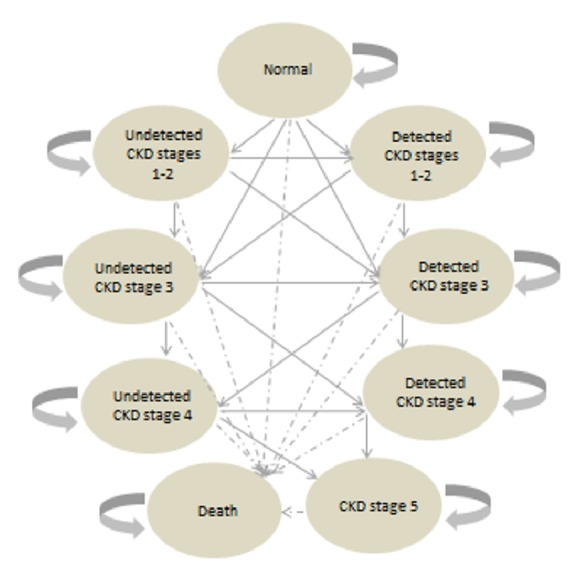
Markov state transition model for the progression of CKD.

In this study, we projected the number of residents with CKD stages 1 to 5 between 2007 and 2035 using a Markov cohort simulation based on a discrete time interval of 1 year (i.e., 28 one-year cycles). We developed a Markov model with nine mutually exclusive health states according to the clinical course of CKD: (i) Non-CKD; (ii) stages 1-2 (undetected); (iii) stages 1-2 (detected); (iv) stage 3 (undetected); (v) stage 3 (detected); (vi) stage 4 (undetected); (vii) stage 4 (detected); (viii) stage 5; and (ix) death (Figure 1).

Transition between health states occurs on a one-year cycle. During the 28 one-year cycles, population growth in each health state occurs through entry of new population (i.e., net migrants and live births) and cohort transition from one health state to another during each cycle. Subjects who start at the “normal” health state may stay in the same state or progress to CKD stages 1-2 (with proteinuria) or CKD 3 (without proteinuria) during the year ahead. If they progress to CKD stages 1-2 and are screened, they will move to the “detected CKD stages 1-2” state in the next cycle. If they progress to CKD stages 1-2 and are not screened, they will move to “undetected CKD stages 1-2” instead. If they do not progress, they will remain at the state of “normal”. Similarly, those who start at “detected CKD stages 1-2” may progress to CKD stage 3. If they were screened, they will move to “detected CKD stage 3” in the next cycle; otherwise, they will move to “undetected CKD stage 3”. However, if they do not progress, they will remain at the state of “detected CKD stages 1-2” regardless of whether screening is performed.

### 2.2. Data Sources

In 2007, the National Healthcare Group (NHG) launched an enterprise-wide chronic disease registry, Chronic Disease Management System (CDMS), to deliver comprehensive and continuous care for patients with chronic diseases [23]. The CDMS links administrative and clinical information of patients who seek care at NHG, which includes three acute care public hospitals and nine primary care clinics. The integrated data enables information access and longitudinal tracking of patient outcomes across different care settings from inpatient, emergency department and specialist outpatient clinics to primary care clinics. Despite the unavailability of private healthcare utilization records, CDMS was able to capture 69% of all detected diabetes and 73% of all detected prediabetes in Singapore [20]. Thus, CDMS is representative of the patient population in Singapore.

Input values for the base year in this study were shown in Table 1.

### 2.3. Definition of CKD in the CDMS

The definition of CKD in CDMS follows the Kidney Disease: Improving Global Outcomes (KDIGO) 2012 CKD Guidelines [25]. In CDMS, earlier CKD stages (stages 1 and 2) are defined based on both estimated Glomerular Filtration Rate (eGFR) level and marker of kidney damage such as Urinary Albumin-to-Creatinine Ratio (UACR) and Urinary Protein-to-Creatinine Ratio (UPCR), whilst moderate to severe CKD stages are defined primarily by eGFR level (Table 2).

For simplicity, patients with eGFR 30-59 mls/min/1.73m2 were defined to have CKD stage 3. The eGFR in the CDMS was estimated using Modification of Diet in Renal Disease (MDRD) equation.

### 2.4. Estimating the Coverage of CDMS for CKD

The national numbers of patients with CKD stages 1 to 3 were computed based on the national prevalence of each CKD stage estimated from a local population-based study [1]. The CDMS coverage for CKD stages 1 to 3 were derived by taking the prevalent numbers of patients in each stage in the CDMS as a proportion of the national numbers of patients in the respective CKD stage.

The yearly national incidence of CKD stage 5 was available from the National Registry Disease Office (NRDO). The CDMS coverage for stage 5 was estimated based on the proportion of stage 5 incident cases in the CDMS divided by the national CKD stage 5 incidence. As the NRDO does not report incidence of CKD stages 1 to 4, the CDMS coverage for stage 4 was estimated by taking the average of CDMS coverage for stages 3 and 5, based on the assumption that the CDMS coverage rises with CKD severity as a result of higher healthcare utilization by those with more severe conditions.

To attenuate the risk of underestimating the CDMS coverage for each CKD stage for base year (i.e., 2007, the same year the CDMS was launched), the CDMS coverage for 2010 was also computed by assuming that the data captured in CDMS would be stabilized after 3 years. The average CDMS coverage of years 2007 and 2010 was estimated for each CKD stage and was used in this study.

### 2.5. National Prevalence of CKD in the Base Year

The prevalence of CKD in 2007 was estimated from a study by Sabanayagam et al. [1] which was a local population-based epidemiological study conducted in 2007. The authors used objective measurements to determine the presence of CKD among multiethnic groups (Chinese, Malay, and Indians) in Singapore. Hence 2007 was used as the base year to build the model in our study.

Sabanayagam et al. [1] reported the national prevalence of CKD stages 1-2, 3, 4, and 5 to be 10.0%, 5.3%, 0.2%, and 0.01%, respectively. CKD stages 1-2 were defined as eGFR ≥60 ml/min/1.73 m^2^ with the presence of albuminuria. However, as the KDIGO defined kidney damage as persistent abnormality in albumin-creatinine ratio or other markers for more than 3 months [25], the use of a single UACR measurement by Sabanayagam et al. [1] to define albuminuria could have overestimated the prevalence of CKD stages 1-2. In this study, we estimated the proportion of individuals with persistent albuminuria among the people with stages 1-2 based on percentages reported in the literature [26–28] and adjusted the pertinent prevalence reported by Sabanayagam et al. [1] to 5.6%.

The prevalence of CKD stages 4 and 5 reported by Sabanayagam et al. [1] was low, as the survey could have been underrepresented by those with severe CKD. Hence, we estimated the respective prevalence using the CDMS coverage. We divided the numbers of prevalent patients in the CDMS by the CDMS coverage to derive the national numbers of individuals with stages 4 and 5 and further divide these numbers by the number of Singapore residents to yield the national prevalence of CKD stages 4 and 5. No adjustment was made to the prevalence of CKD stage 3 reported by Sabanayagam et al. [1].

### 2.6. National Prevalence of Detected CKD in the Base Year

For simplicity, we assumed the coverage of the CDMS for detected CKD to be 70%, similar to the rate for detected diabetes (69%) and prediabetes (73%) at national level [20]. We derived the national numbers of detected CKD stages 1 to 4 based on this assumption and the respective numbers of prevalent CKD patients in the CDMS. Further comparison of the national numbers of detected CKD stages 1 to 4 with the respective national numbers of CKD (both detected and undetected) yielded the detection rates for the four stages. We multiplied the CKD prevalence rates with detection rates to obtain the prevalence of detected CKD for each stage.

### 2.7. Annual Transition and Detection Probabilities

Movement of individuals between health states over time was tracked using transition probabilities. The historical transition probabilities of each CKD stage were derived primarily from the CDMS based on the annual incidence of each CKD stage. Historical CKD detection probabilities were determined by specifying an equation representing sources of detected CKD incident cohorts and equated this equation with the historical observed data. Detection probabilities from this equation were derived based on the assumption that ratio of detection probabilities of individuals at a higher (e.g., CKD stage 3) to lower (e.g., CKD stages 1-2) disease continuum was equal to their mortality rate ratio.

To forecast future time-dependent transition and detection probabilities between 2015 and 2035, we computed the base transition and detection probabilities by averaging the respective probabilities from 2010 to 2014 and elevated these base probabilities for CKD stages 1 to 4 using an ageing index. The ageing index is a product of the yearly proportion of elderly residents (aged 65+ years) and the yearly increase in the proportion of the elderly from the reference rate (5-year average elderly proportion in 2010-14). Elevation of transition and detection probabilities using the aging index aims to address the forthcoming population ageing in Singapore, given that kidney function declines with ageing [19] and the elderly has higher healthcare utilization [29], thus translating into higher CKD incidence and detection rates.

The ageing index was not applied to the transition probabilities from stage 4 to stage 5 as it was found that there were higher competing risks of death among the elderly patients [30, 31]. For stage 5, we assumed 100% detection rate in this study as the NRDO captures the incidence of renal failure at national level.

### 2.8. Historical and Forecast Mortality Rates

The mortality risks of individuals with CKD were found to be higher than those without CKD [32]; thus we assumed the mortality rate of individuals with CKD was equal to the product of the relative risk of death for people with CKD and the mortality rate of those without CKD as in the following equation:

(1) $$\begin{array}{c} {MR}_{it}={RR}_{it}\times {MR}_{t} \end{array}$$

where MR_it_ is the mortality rate of individuals with CKD stage* i* at time* t*; MR_t_ is the mortality rate of individuals without CKD at time* t*; RR_it_ is the relative risk of death for individuals with CKD stage* i* versus without CKD at time* t*

#### 2.8.1. Historical Mortality Rates

For individuals with CKD, we estimated their historical mortality rates at different CKD stages (ie. MR_it_) using both hospital deaths and national deaths recorded in the CDMS. For those without CKD, the historical mortality rate at time* t *(i.e., MR_t_) was derived using (2), where the historical number of deaths at time* t* at national level (i.e., MV_t_) was available from public source [33]:

(2) MVt=MR12t×CKD12t+MR3t×CKD3t+MR4t×CKD4t+MR5t×CKD5t+MRt×NCKDt

where MV_t_ is the total number of deaths at time* t*; MR_it_ is the mortality rate of individuals with CKD stage* i* at time* t*; MR_t_ is the mortality rate of individuals without CKD at time* t*; CKD_it_ is the estimated number of individuals with CKD stage* i* at time* t*; NCKD_t_ is the estimated number of individuals without CKD at time* t.*

Based on MR_it_ and MR_t_, we derived historical RR_it_ using (1) and estimated the 5-year average (2010 to 2014) relative risk of death for individuals with CKD stage* i* versus without CKD (i.e., RR_i_) based on RR_it_.

#### 2.8.2. Forecast Mortality Rates

Future mortality rates of individuals without CKD were forecast based on (3). Detailed methodologies on the forecasts of national residents and residents' mortality (i.e., MV_t_) have been published elsewhere [20]. In short, we projected future population growth using compounded annual growth rates and forecast future MV_t_ for those aged 21+ years at national level after we forecast (i) life expectancy; (ii) number of deaths of all ages; and (iii) number of deaths of people aged <21 years.

(3) $$\begin{array}{c} MR_{t}=\frac{MV_{t}}{\left(RR_{12}\times CKD_{12t}+RR_{3}\times CKD_{3t}+RR_{4}\times CKD_{4t}+RR_{5}\times CKD_{5t}+{NCKD}_{t}\right)} \end{array}$$

The forecast mortality rates of those without CKD were used as input values to forecast mortality rates of individuals with CKD using the following equation:

(4) $$\begin{array}{c} MR_{it}=RR_{i}\times MR_{t} \end{array}$$

### 2.9. Other Assumptions

We assumed individuals will reside in a health state for a minimum of one cycle (i.e., 1 year) before progressing to the next and the CKD progression was one-way without regression to the previous state.

### 2.10. Approval

Approval to conduct this study was obtained from the NHG Ethics Review Board (Domain-Specific Review Board).

## 3. Results

### 3.1. Forecasting the Prevalence and Number of CKD Individuals

**Figure 2:**
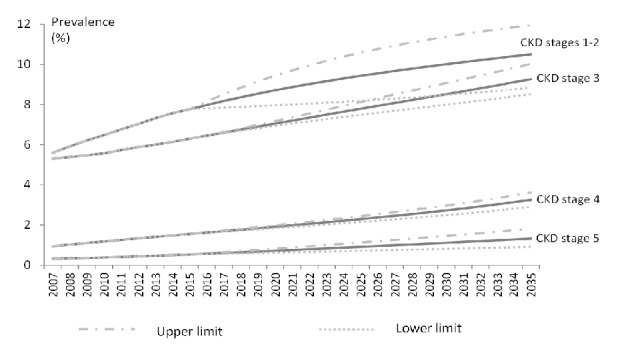
Prevalence of CKD by stage, with projection till 2035.

After adjustments to CKD prevalence using estimates from Sabanayagam et al. [1], we estimated there were 145,803, 137,992, 24,293, and 8,434 cases of CKD stages 1-2, 3, 4, and 5, respectively, in 2007. By 2035, the Markov model projected the numbers to be 383,122 (95% CI: 322,402 - 435,781), 337,779 (95% CI: 310,663 - 365,407), 118,821 (95% CI: 106,048 - 131,950), and 48,148 (95% CI: 33,271 - 66,070), respectively. The total number of individuals with CKD in 2035 is almost triple that in 2007 and the prevalence is projected to increase from 12.2% in 2007 to 24.3% (95% CI: 21.2% - 27.4%) in 2035 (Figure 2). Throughout the 28 years, stages 1-2 constituted the largest proportion of CKD cases, followed by stages 3, 4, and 5. During this period, the proportion of people with stages 4 and 5 is estimated to increase from 7.7% to 13.4% and from 2.7% to 5.4%, respectively. Number of individuals with stage 5 is expected to increase by 5-fold from 8,434 in 2007 to 48,148 in 2035.

**Figure 3:**
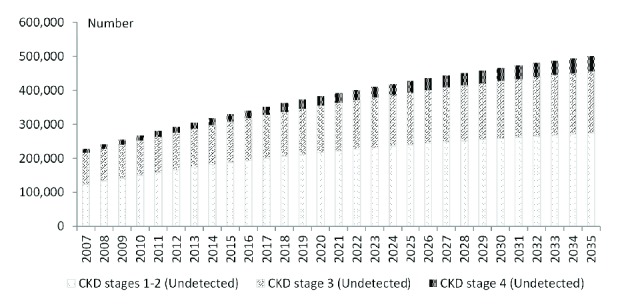
Number of undiagnosed cases of CKD by stage, with projection till 2035.

The overall prevalence of undiagnosed CKD was 8.8% (stages 1-2: 4.8%; stage 3: 3.5%; stage 4: 0.4%) in 2007. This is projected to increase to 13.7% (stages 1-2: 7.5%; stage 3: 5.0%; stage 4: 1.2%) by 2035. The proportion of undiagnosed cases is expected to decline from 72.1% in 2007 to 56.4% in 2035. The increase in the detection rates is postulated to be fuelled by the faster progression to the higher CKD stages as part of population ageing. As older patients and those with higher CKD stages are more likely to have routine visits to healthcare, we expect them to have laboratory tests as part of their regular care thus increasing the likelihood of detecting CKD. Throughout the 28 years, CKD stages 1-2 remained the main sources of undiagnosed cases as these contributed more than half of such cases (Figure 3).

### 3.2. Sensitivity Analysis

The sensitivity analysis showed that an annual 1% reduction in the incident CKD stages 1-2 between 2007 and 2035 could prevent 5,197 CKD cases from the baseline forecast of 887,870. Similarly, an annual 1% increase in detection rate for stages 1-2 would reduce 942 undetected CKD cases over baseline forecast, whilst an annual 1% increase in detection rate for stage 4 would reduce 208 cases. Thus, a steady 1% increase in detection rate annually for CKD stages 1-2 vis-à-vis CKD stage 4 would reduce undetected cases by 734.

## 4. Discussion

This study projected that the number of individuals with CKD will reach 887,870 in 2035, almost triple that in 2007, whilst the number of undiagnosed CKD cases is forecast to be 500,600, more than double that in 2007. One of the main drivers behind the surge in CKD prevalence could be the increase in incidence of diabetes and hypertension. In Singapore, the prevalence of diabetes and hypertension in 2010 was 11.3% and 23.5%, respectively [34]. This represents 56% and 7% increases in the numbers of people with diabetes and hypertension, respectively, over 6 years from 2004 [34]. By 2035, the prevalence of diabetes among Singapore residents is projected to be 1 in 5 [20]. Although the forecast of the prevalence of hypertension is unavailable, it is expected to rise in tandem with that of diabetes as both conditions share common pathophysiologic pathways [35]. Apart from this, population growth and the longer life expectancy of Singapore residents are other potential drivers leading to increase in the prevalence of CKD. The life expectancy at birth in Singapore had increased steadily from 65.8 years in 1970 to 82.7 years in 2015 [33]. The upward trend is expected to continue and projected to reach 87.7 years by 2035 [20]. The ageing population is postulated to increase the CKD cases as renal impairment is common in the elderly.

Previous literature [36, 37] raised concerns on the use of a universal GFR threshold of 60 ml/min/1.73m^2^ to define CKD, in particular among the elderly, as decline in “normal” GFR with ageing in the absence of kidney damage marker is physiologic and the associated mortality risk of those with eGFR of 45-59 ml/min/1.73m^2^ was found to be trivial. Various suggestions had been made in the literature to revise the CKD classification, including lowering the eGFR threshold to below 45 ml/min/1.73m^2^ for stage 3 definition [38] and introducing age- and gender-specific qualifying levels of GFR [39, 40]. If we revised our projection by excluding the elderly aged 65+ years whose eGFR was 45-59 ml/min/1.73m^2^ but without proteinuria, the adjusted number of CKD individuals in 2035 is estimated to be reduced by one-quarter from the baseline forecast of 887,870 to 668,987. This study used MDRD equation to define CKD stage. Our forecast of CKD numbers may be different if CKE-EPI was used as it more accurately estimates GFR and categorized ESRD risk than the MDRD equation [41]. CKD-EPI classified fewer individuals as having CKD [41] and thus our forecast of CKD prevalence would be lower if CKD-EPI was used. Nevertheless, we are unable to estimate the magnitude of the reduction as identification of individuals with CKD using CKD-EPI is currently not configured in the CDMS.

With the expected rise in CKD in the coming years, more extensive health resources including ambulatory, hospitalization, and dialysis care would be required. In Singapore, the prevalence of ESRD increased from 1,405 per million residents in 2006 to 2,076 per million residents in 2016, representing 47.8% increase in a decade [42]. Our healthcare expenditure had increased from 4% of GDP expenditure in 2005 to 4.9% in 2014 [43]. The health system would need to be prepared for the significant surge in demand for health services in the next decades. However, CKD is often undiagnosed, largely due to its asymptomatic nature. Our CKD detection rate in the base year was low at 27.9%. In countries with the highest ESRD incidence rates in the world such as USA, Thailand, and Taiwan, the CKD awareness rates among those with CKD were even lower (USA: 6% [44]; Thailand: 1.9% [45]; Taiwan: 3.5% [5]). In USA, despite the efforts to increase CKD awareness among the nephrologists, general physicians, and the public via dissemination of KDOQI guidelines, setting up of CKD education programmes, and offering free screening to the public, there was merely marginal improvement in awareness rates [44]. In Thailand, the low awareness could be attributed to the underdiagnosis of CKD as only serum creatinine was widely available and used by the local healthcare professionals to assess kidney function, instead of eGFR prediction equation such as MDRD equation [45]. Similarly, the eGFR prediction equation based on calibrated creatinine was not commonly used in Taiwan, leading to underdiagnosis of CKD and low awareness among the patients [5]. Our detection rate was relatively high compared to the awareness rates in USA, Thailand, and Taiwan. This could be due to the fundamental differences in definitions and methodologies used to estimate the CKD detection and awareness rates. Whilst our CKD detection rate was derived from the CDMS using MDRD prediction equation, the awareness rates reported by the overseas studies [5, 44, 45] were ascertained from patient surveys using generic questions such as whether they had ever been told to have (i) kidney disease in general (which could have included urinary tract infection or urinary stones) [5] or (ii) weak or failing kidneys (excluding kidney stones, bladder infections, or incontinence) [44]. Both the low detection rate in our study and the low awareness rates reported by the overseas studies [5, 44, 45] suggest that underdiagnosis and lack of awareness of CKD are common global issues. As CKD is treatable, low disease awareness and detection would need to be addressed. The use of equations for prediction of eGFR by healthcare professionals needs to be encouraged to increase the detection of CKD cases.

Delaying the progression of CKD to later stages, or even primary prevention of CKD, is possible through pharmacological intervention or lifestyle modification. Lifestyle modification [46] such as having regular physical activity, healthy diet, BMI≤25, moderate or less alcohol consumption, and being a nonsmoker are beneficial for primary prevention of CKD, possibly through the prevention of diabetes and hypertension as the three conditions have common pathway in disease development. Combined effect of the healthy lifestyle factors is reported to significantly reduce risks of cardiovascular diseases and CKD, and there was a dose-response relationship between the number of healthy lifestyle factors attained and magnitude of disease risk reduction [46]. Thus, leading a healthy lifestyle could play a major role in the war against CKD. For individuals with CKD, pharmacological intervention, such as the use of inhibitors of the renin-angiotensin system to control hypertension and proteinuria, has also been found to be effective in delaying the CKD progression [47]. Regression of proteinuric CKD is achievable particularly in patients without diabetes [48]. Ricardo et al. found that, among persons with CKD, nonsmokers and BMI≥25 were associated with lower risk of CKD progression, whilst having regular physical activity, nonsmoking, and BMI≥30 were associated with reduced all-cause mortality. In the general population, elevated BMI is associated with an increased risk of cardiovascular events; however, in individuals with CKD, BMI was found to have an inverse relationship with CKD progression or mortality [49, 50]. Reasons for the paradoxical association are unclear to date; some proposed explanations included higher BMI signaled nutritional adequacy [51] and more stable hemodynamic status [52]. Recently, there is emerging evidence on the role of diet in kidney health. Snelson et al. [53] reported that imposing dietary constraints and optimizing diet quality could complement therapies for CKD prevention or retarding CKD progression, as diet is implicated in the kidney health via modification of gut homeostasis or through haemodynamic effects [53]. Currently, patients with CKD are recommended to restrict sodium, potassium, and protein intake [53]. Whilst McMahon et al. [54] reported that restriction on salt consumption in patients with later CKD stages was effective for reducing blood pressure, albuminuria, and proteinuria, Adrogué et al. [55] found that the salt-sensitivity in patients with CKD might be abolished by the consumption of diet high in potassium which is believed to be antihypertensive, thus slowing the progression of CKD to later stages. There is also emerging evidence [53] that maintaining protein balance with adequate prevention of protein energy wasting by shifting from animal to plant-based protein intake may improve renal outcomes. More studies are needed before changes are made to the current recommendations.

Our study was limited by the lack of demographic specific national prevalence of detected CKD at base year and the forecast mortality rates. Thus we could not stratify the projections of individuals with CKD by subpopulations. This may underestimate the disease burden especially with the expectant ageing population in Singapore. To overcome this problem, we used an ageing index to elevate the transition and detection probabilities to compensate for the effect of population ageing. Although this might not fully capture the complexities of age on the changes in the transition and detection probabilities, elevation of the two probabilities using the ageing index could partially address the forthcoming population ageing that may potentially cause a rise in number of individuals with CKD.

This study assumed that the CKD progression was one-way without regression to the previous state. However, mild CKD, in particular CKD stages 1 and 2, is reversible. Previous study [56] found that 30%-54% of individuals with diabetes had regressed from moderate albuminuria to normo-albuminuria. Our assumption on one-way disease progression will inevitably result in overestimation of the number of individuals with more advanced CKD state in 2035. This study did not model the progression of each CKD stage based on the presence or absence of proteinuria due to incomplete data. As proteinuria is a strong predictor of an increased risk of disease progression [57], the lack of complete data signals that our transition probabilities estimated from the CDMS for each CKD stage are biased by sampling variations. Our projections could also be affected by future migration trends as susceptibility to diseases among the foreign-born residents could be different from the local population and these would impact the stability of the disease transition probabilities. Future ethnicity ratio is unlikely to significantly affect our results as the ethnic distribution in Singapore is likely to remain stable in the next two decades.

Despite these limitations, this study was conducted using robust methodology. We simulated the disease progression based on time-dependent probabilities and rates and systematically projected the number of individuals with CKD in each stage according to the clinical course. We estimated the number of undiagnosed cases to quantify the magnitude of the disease burden so that early interventions could be formulated. Whilst our findings may be artifacts of the projection methodologies, we are unaware of any local CKD study that can provide evidence to support or contradict our projections and findings. To the best of our knowledge, this is the first study on the projection of number of individuals with CKD in Singapore, with insights into undiagnosed CKD burden. Thus further research is warranted.

By 2035, about one-quarter of the Singapore residents aged 21+ years are expected to have CKD. Of these, more than half remain undiagnosed and majority of these undiagnosed cases are contributed by the CKD stages 1-2. National policies need to focus on primary disease prevention and early disease detection to avoid delayed treatment of CKD leading to ESRD. The forecast of future burden of CKD and the number of undiagnosed cases in this study can aid in the planning of future healthcare resources and manpower, which in turn translates into improvement in the healthcare system, better preventive care, and favourable patient outcomes.

## Figures and Tables

**Table 1:** Input values for the base year.

Parameter (aged 21+ years)	Value	Source(s)
**Demographic variables**		
Prevalence of non-CKD, 2007 (unadjusted)	84.4%	Sabanayagam et al., 2010 [1]

Prevalence of CKD stages 1-2, 2007 (unadjusted)	10.0%	Sabanayagam et al., 2010 [1]

Prevalence of CKD stage 3, 2007 (unadjusted)	5.3%	Sabanayagam et al., 2010 [1]

Prevalence of CKD stage 4, 2007 (unadjusted)	0.2%	Sabanayagam et al., 2010 [1]

Prevalence of CKD stage 5, 2007 (unadjusted)	0.01%	Sabanayagam et al., 2010 [1]

% undiagnosed CKD stages 1-2, 2007 (adjusted)	85.3%	Sabanayagam et al., 2010 [1] and CDMS (with assumption)

% undiagnosed CKD stage 3, 2007 (adjusted)	66.9%	Sabanayagam et al., 2010 [1] and CDMS (with assumption)

% undiagnosed CKD stage 4, 2007 (adjusted)	47.3%	Sabanayagam et al., 2010 [1] and CDMS (with assumption)

**Singapore resident population**		
Singapore resident population, 2007	2,603,628	Population Trends 2014 [24]

**Mortality rates (per 1000 resident population)**		
Mortality rate, 2007	6.50	Population Trends 2014 [24]

CDMS: Chronic Disease Management System; CKD: Chronic Kidney Disease.

**Table 2:** Definition of CKD in the CDMS.

**Stages of CKD**	**eGFR**	**Marker of kidney damage in CDMS**
Early (Stages 1-2)	≥ 60 ml/min/1.73 m^2^	Any 1 of the following: (i) Two UACR lab tests ≥ 2.5 mg/mmol (male), 90 days apart (ii) Two UACR lab tests ≥ 3.5 mg/mmol (female), 90 days apart (iii) Two UACR lab tests > 30mg/g, 90 days apart (iv) Two UPCR lab tests ≥ 20mg/mmol, 90 days apart (v) Two UPCR lab tests > 0.2mg/mg, 90 days apart (vi) Two Urine Protein lab tests ≥ 0.2 g/day, 90 days apart

Moderate (Stage 3A)	45-59 ml/min/1.73 m^2^	With or without kidney damage

Moderate (Stage 3B)	30-44 ml/min/1.73 m^2^	With or without kidney damage

Severe (Stage 4)	15-29 ml/min/1.73 m^2^	With or without kidney damage

Severe (Stage 5)	<15ml/min/1.73 m^2^	With or without kidney damage

## Data Availability

The registry data used to support the findings of this study cannot be made available because data sharing is restricted by the Domain-Specific Review Board in the National Healthcare Group in order to protect patient privacy.
